# Iron- and protein rich diets may boost hemoglobin levels among informal electronic waste recyclers exposed to metals at Agbogbloshie, Ghana

**DOI:** 10.1016/j.heha.2023.100073

**Published:** 2023-07-10

**Authors:** Sylvia A. Takyi, John Arko-Mensah, Niladri Basu, Serwaa Bawuah, Duah Dwomoh, Julius N. Fobil

**Affiliations:** a School of Public Health, University of Ghana, Legon. Accra Ghana; b McGill University, Montreal, QC, Canada

**Keywords:** E-waste, Metals, Diet, Hemoglobin, Anemia, Agbogbloshie

## Abstract

While human exposure to metals may play a role in the pathogenesis of anemia, consumption of balanced diets may boost blood hemoglobin (Hb) levels in humans. Although informal electronic waste (e-waste) recycling processes have recently drawn attention as an important source of pollution, there is almost no empirical evidence on the relationship between diet, metals exposure and anemia among e-waste recyclers. Therefore, we evaluated possible ameliorating effects of diet on metal exposure related anemia, as measured by Hb levels of e-waste recyclers and a reference population in Ghana.

This repeated measure study used data collected from e-waste recyclers (*n*=142) and a reference population (*n*=65) between March 2017 and October 2018. Stored whole blood samples were analyzed for the following metals; Cd, Pb, Rb, Tb, Tl, and Eu. Next, Hb levels were analysed using the URIT-810^®^ semiautomatic biochemistry analyzer. Furthermore, a 48-hour dietary recall questionnaire was administered to assess dietary intake parameters such as protein, folate, carbohydrates, Fe, Ca, Mg, Se, Zn, and Cu. Ordinary regression models were used to estimate joint effects of metals and nutrients on Hb levels.

At baseline, the mean Hb was lower among recyclers (12.99 ± 3.17 g/dL) than the reference group (13.02 ±2.37 g/dL). Blood Pb, Cd, Rb, Eu and Tb were associated with significant decreases in Hb levels of e-waste recyclers. Dietary intake of proteins and Fe was associated with concomitant increase in Hb levels of both groups as well as when analysis was restricted to recyclers. Despite the high exposure of e-waste recyclers to a myriad of metals, consumption of Fe-rich diets appears to ameliorate anemia and improved Hb levels (β=0.229; 95% CI: 0.013, 0.445; *p*=0.04). Therefore, the consumption of Fe and protein-rich foods may boost blood Hb levels in e-waste recyclers, even though exposure to high levels of metals is a predictor of anemia among this worker-group.

## Introduction

Human exposure to metals has become more widespread in recent decades, owing to their ubiquity in the environment. In low and middle-income countries (LMICs) today, it is widely believed that the recycling of electronic waste is a significant contributor of metal discharge into the ambient environment. In and around sites that e-waste recycling occurs, there is considerable discharge of metals into the environmental media as revealed by research conducted in Ghana, China and other parts of the world because of the use of crude recycling methods such as open-air burning, dismantling, collection and sorting (Takyi et al., 2021; Srigboh et al., 2016; Carlson et al., 2021). The adoption of such inappropriate techniques potentiates the release of chemicals (e.g. metals, organic compounds and particulates) into the environment. In particular, the exposure to metals such as Cadmium (Cd), Lead (Pb), strontium (Sr), Thallium (Tl)) has been reported to be associated with endocrine disruption and metabolic disorders (Engwa et al., 2019; Anyanwu et al., 2018; Rehman et al., 2018; Paschoalini et al., 2019; Piscopo et al., 2020).

For some years now, adverse health outcomes linked to chemical exposures associated with informal recycling of e-waste have been under intense research focus by many research groups (Kamal and Malik, 2012) around the globe. For example, chronic exposure to Pb released from e-waste recycling activities may impair hemoglobin (Hb) production by interfering with enzymatic pathway in the heme synthesis, resulting in a reduction in red blood cells (RBC) and anemia (Abadin et al., 2007; Hsieh et al., 2017; Kutllovci-Zogaj et al., 2014; R Rahimpoor et al., 2020). Other researchers have also linked environmental exposure to Pb and Cd with anemia (Hsieh et al., 2017; Camaj et al., 2020); including interference with the mechanisms of erythrocyte survival (Hernberg et al., 1967; Leikin and Eng, 1963), defective erythropoiesis (Peters et al., 2021; Sassa, 1978) and inhibition of enzymatic pathway for heme synthesis and metabolism (Moore et al., 1987; Balali-Mood et al., 2021; Trombini et al., 2015; Mukisa et al., 2020).

Of note, nutritional factors and diets are able to modify mechanistic pathway in the development of anemia linked to human exposure to toxic metals. For instance, while Pb exposure may induce anemia in humans by interfering with the mechanism of heme synthesis, adequate consumption of iron (Fe) and vitamin C rich diets has been found to have beneficial effects ameliorating heme synthesis (23). Furthermore, Vitamin A, for example, stimulates RBC synthesis and promotes immunity by mobilizing Fe reserves from tissues, and as a consequence, contributes to lowering infection-related anemia (Semba and Bloem, 2002; Tata et al., 2019).

In this study, we investigated the relationship between exposure to metals and hemoglobin levels and how this relationship is mediated by nutritional and dietary factors.

## Material and methods

### Study design

We analyzed archived blood samples, stored for approximately five years in −80 °C, which was collected during a longitudinal study; with three repeated rounds, for Hb levels in male e-waste recyclers and a reference population located approximately 18 km away from the e-waste recovery site at Agbogbloshie (Takyi et al., 2021; SA Takyi et al., 2020; SA Takyi et al., 2020). Women and children were not included in this study as planned Geo-Health-II longitudinal cohort study. It is worth noting that most e-waste recyclers in Agbogbloshie, as well as a significant portion of the reference population in the Madina-Zongo area, were internal migrants who periodically engaged in subsistence farming in the northern region of Ghana, thus were a highly mobile population.

The time-points for the repeated measures (time I [March–April 2017] (dry season), time II [July–August 2017] (rainy season), and time III [March–April 2018] (dry season)). were spaced-out to allow for the analysis of seasonal variability in exposure dose (Takyi et al., 2021). Nutrition data collection spanned one to two weeks for each time point. We used a semi-structured 2-day 24-hour recall guide to gather information on the daily nutritional intake of participants. This guide was administered twice to estimate the day-to-day variability per individual, as the variety of foods consumed on different days can significantly impact nutritional intake. Our interview consisted of foods and beverages (e.g., the amount, time, and types) consumed on one weekday and 1 day of a just past weekend (Saturday or Sunday). During time I, a total of 142 e-waste recyclers and 65 non-e-waste recyclers (reference group) were interviewed face-to-face (SA Takyi et al., 2020; SA Takyi et al., 2020). This allowed us to investigate the association between metal exposure and anemia and how this association is influenced by diet and nutritional factors.

### Archived whole blood sample analysis

#### Elemental analysis

Blood level concentrations of metals; namely, Cadmium (Cd), Lead (Pb), Strontium (Sr), Cerium (Ce), Rubidium (Rb), Yttrium (Y), Europium (Eu), Lanthanum (La), Neodymium (Nd), Thallium (Tl), and Terbium (Tb) were measured in stored whole blood samples. Inductively Coupled Plasma Mass Spectrometer (ICP-MS; Varian 820MS, Analytik Jena, Germany) was used to measure the elemental (toxic metals and micronutrients) levels). Before use, all tubes and pipette tips were acid-washed (cleaned, soaked 24 h in 10% hydrochloric acid, and then rinsed three times in Milli-Q water).

On day one, 15 mL digitube vials for sample digestion were labeled with special codes. Next, each, whole blood sample was thawed and vortexed for about six minutes on a tabletop vortex machine. Following this, 200 μl of blood and 400 μl of 70% nitric acid were sampled into 15 mL digitubes vials and lightly capped. The aliquots were allowed to rest overnight in a fume hood at room temperature. The next day, 200 μl of 30% Hydrogen Peroxide was added to the vials, loosely capped, and allowed to rest overnight in a fume hood at room temperature. On the third day, 1.2 ml of milli-Q water was added to each vial and kept prior to sample analysis. Finally, 4.375 ml of milliQ water, 0.625 ml of 10ppb gallium (Ga) solution, and 1.25 ml of the blood digest were aliquoted into 10 ml pre-label borosilicate auto-sample vials for analysis. The samples were then ready for ICP-MS metal analysis.

#### Hemoglobin (Hb) analysis

Blood hemoglobin (Hb%) levels were determined using a “cyanmethemoglobin method. First, the Drabkin’s solution was prepared from potassium ferricyanide, potassium cyanide, and sodium bicarbonate. Next, 20 microliters (μL) of blood was mixed with the 5.0 ml of Drabkin’s reagent and allowed to stand for 5 min to develop color (Pintavirooj et al., 2021; Madani et al., 2015). The resulting intensity was measured against a known standard at 546 nm using a URIT-810^®^ semiautomatic biochemistry analyzer (Thermo Corp., Cambridge, UK) (Bashir and Derar, 2019). An average of three Hb readings was taken for each blood sample analysed. Average levels of Hb in each sample were then calculated as [A546_unk_ × Hb Standard concentration (g/dl)]/A546_std_.

The World Health Organization (WHO), anemia is defined as hemoglobin (Hb) levels <13.0 g/dL in men (Cappellini and Motta, 2015; Kim et al., 2019). Accordingly, a cut-off point of < 13.0 g/dL was used to diagnose anemia in male e-waste recyclers and reference groups in this study.

### Data analysis

#### Nutrient analysis

As published by our research team, nutrient intake data was converted to grams using Ghanaian Food Composition Tables (SA Takyi et al., 2020; SA Takyi et al., 2020). Furthermore, nutritional analysis was performed using the ESHA F Pro-software^®^ to estimate individual nutrient intake. Following the nutritional analysis, data acquired from the ESHA F Pro-included amounts of proteins, vitamin B12, folate, calcium (Ca), magnesium (Mg), iron (Fe), zinc (Zn), copper (Cu), and selenium (Se) consumed from food. In addition, the mean probability of micronutrient adequacy was calculated to determine the percentage of individuals who fulfilled the Recommended Daily Allowance (RDA) for adult males overtime time (Mahan and Raymond, 2016).

### Statistical analysis

#### Comparison of blood metal levels and Hb overtime

At each time point, the Welch *t*-test was used to compare the actual mean distribution of blood metal levels measured between e-waste recyclers and the reference population. The Welch’s *t*-test was used since the variances of the outcome measures were not equal among the e-waste recyclers and the reference population. Similarly, Hb levels were analysed using the Welch’s *t*-test. Sociodemographic characteristics reported in our recent publications (Takyi et al., 2021; SA Takyi et al., 2020; SA Takyi et al., 2020; Amoabeng Nti et al., 2020) are also controlled for in the current paper. These characteristics include age, daily income earnings, cigarette smoking, biomass burning, marital status, work duration and religion.

#### Nutrient (Macro and micronutrient intake) by e-waste recyclers and reference population

As detailed in earlier publications (SA Takyi et al., 2020; SA Takyi et al., 2020), the z-test was used to compare proportions of e-waste recyclers and the reference group who met the Recommended Dietary Allowance (RDA) for nutrients. The current study similarly compared the proportion of recyclers and reference group who met the RDA for dietary protein intake as well as folate and vitamin B12. This comparison was conducted by categorizing each of the measured nutrients according to the United States Department of Agriculture (USDA) recommendations for adults [58, 59]. The USDA definition describes the nutrient adequacy based on data obtained from participants’ reporter nutrient intake. The Welch *t*-test was used to compare the actual mean distribution of nutrient intake between e-waste recyclers and reference population at each wave. Given that the variances of the outcome measures were not equal across the e-waste recyclers and reference group, the Welch’s *t*-test was performed.

#### Effects of metal exposure on nutritional status overtime

A multivariate linear regression model with a robust standard error that adjusted for confounders was used to investigate the relationship between metal exposure and dietary nutrient intake in this study. The covariates controlled for were age, daily income earnings, cigarette smoking, biomass burning, marital status, work duration and religion.

#### Effects of metal exposure on hemoglobin (Hb) levels overtime

The regression model above was further used to evaluate the relationship between metal exposure and hemoglobin levels.

#### Joint effect of metals and nutrients on Hb levels overtime

The Shapiro Francia test was used to examine the normality of the continuous outcome measures in the study (Ahad et al., 2011). Prior to performing further statistical analysis, non-normal outcome measures were log-transformed. Next, we used the Hausman’s test to guide us decide between the fixed and random-effects models. The fixed-effects model was eventually used to assess the joint effect of metal exposures and diet on Hb levels based on the findings of the Hausman’s test (*p < 0.05*). All statistical tests were performed using the Stata^®^ version 15 software (StataCorp, College Station, Texas, USA).

## Results

### Sociodemographic characteristics of participants

As recently published by our research team (Takyi et al., 2021; SA Takyi et al., 2020; SA Takyi et al., 2020; Amoabeng Nti et al., 2020), the study participants had a mean age of 27.6 ( ± 0.4) years with a range of 18 to 50 years. The e-waste recyclers were less educated, with a quarter of individuals having received no formal education. This is in contrast to the reference population, where over half of them had completed at least senior high school or higher education. The recyclers said they worked for roughly 9 h each day and had been in the e-waste industry for about 10 years. More than half of the recyclers slept on-site, specifically in their working shed at the recycling facility. Moreover, half of the e-waste recyclers reported earning between 20 and 100 Ghana Cedis per day (about $4 to 17 USD per day), with the remaining 24% earning less than 20 Ghana Cedis per day.

#### Metal levels in blood of e-waste recyclers and comparison group overtime

We already published baseline levels of Cadmium (Cd), Lead (Pb), Strontium (Sr), Cerium (Ce), Rubidium (Rb), Yttrium (Y), Europium (Eu), Lanthanum (La), Neodymium (Nd), Thallium (Tl), and Terbium (Tb) in blood (Takyi et al., 2021). Here we report metal levels measured in the two subsequent time points to ascertain seasonal variations of metal exposures overtime (Table 1 & Appendix A).

Following our baseline report, the mean blood levels of Pb, Sr, and Tl among e-waste recyclers remained considerably higher than in the reference population (Table 1a). Mean blood levels of other elements (for example, Ce, Y, La) were greater in the reference group than in the recyclers. Over the course of a year, while levels of Pb, Mn, and Rb in recyclers decreased, the levels of other metals, specifically, Ce, Y, La, Nd, and TI increased. Despite the fact that the levels of Ce, La, and Tl had increased dramatically in the second and third time points, the levels of Pb, Cd, Mn, Rb, Eu, and Tb in the blood rather decreased significantly for the reference population (Table 1b).

#### Nutrient (macro & micronutrient) intake of e-waste recyclers and reference population overtime

As described in a previous publication (SA Takyi et al., 2020), the baseline mean levels of Fe, Mg, and Zn in the diet of e-waste and non-e-waste recyclers differed considerably; the dietary Fe (t (Takyi et al., 2021) =2.70, *p* = 0.004) and Zn (t (Takyi et al., 2021) = 2.81, *p* = 0.01) consumption was significantly greater in e-waste recyclers than in the reference population. Mg intake, on the other hand, was significantly higher among members of the reference population (*p < 0.05*). Furthermore, nearly all study participants consumed acceptable quantities of Fe (SA Takyi et al., 2020), proteins and carbohydrates in diet in accordance with the RDA from March 2017 to April 2018 (Table 2). Significant increase in dietary Fe [β = 0.233; 95% CI: 0.046, 0.420; *p* = 0.02] and Se [β = 0.193; 95% CI: 0.103, 0.282; *p* = 0.01] intake was observed overtime, particularly among recyclers (SA Takyi et al., 2020). The consumption of Ca, Cu, Se, Mg (SA Takyi et al., 2020) as well as vitamins B_12_, A, C, D and folate through diet was generally inadequate in both study groups over time, as per the set RDA guidelines (SA Takyi et al., 2020). Furthermore, we did not observe changes in dietary intake of folate, vitamin A, B_12_, C, D proteins and carbohydrates, during the period of March 2017 to April 2018) (Appendix B).

Table 2 below compares other self-reported dietary intake of micronutrients (iron, folate, vitamins A, B_12_, C and D) and macronutrients (proteins and carbohydrates) between the e-waste recyclers and the reference population.

#### Anemia prevalence

Although the mean Hb levels were generally higher among individuals from the reference population, the mean Hb level of the e-waste recyclers was 14.72 g/dL (Appendix C) and was within the criteria (14–18 g/dL) set for males by the WHO. Significant changes in Hb levels were observed overtime for both e-waste recyclers (β = 0.086; 95% CI: 0.052, 0.119; *p < 0.01*) and the reference population (β =0.068; 95% CI: 0.010, 0.12; *p* = *0.02*). Although not statistically significant, the prevalence of anemia was higher among the recyclers (53%) compared to the reference group (47%) (Table 3). Meanwhile, this prevalence declined overtime among both the recyclers and among the reference population. Comparison within the recyclers groups showed that Hb levels were highest among the burners, followed by the dismantlers and then the sorters/ collectors (Appendix D). We did not observe any significant effect modification by age, daily income earnings, cigarette smoking, biomass burning, marital status and religion.

### Association between nutrient intake and hemoglobin

Dietary intake of Fe-rich foods was found to be associated with significant increase in Hb (β = 0.208; 95% CI: 0.051, 0.366; *p* = *0.01*) levels in both groups (Table 4) as well as when analysis was limited to only recyclers (β = 0.266; 95% CI: 0.066, 0.466; *p* = *0.01*) (Appendix E). Similarly, protein-rich diet intake was associated with significant increase in Hb levels in both groups (β = 0.155; 95% CI: 0.002, 0.309; *p* = *0.04*), and when the analysis was restricted to the recyclers (β = 0.212; 95% CI: 0.032, 0.391; *p* = *0.02*) only. Meanwhile, no significant differences were observed between nutrient intake and Hb levels among members of the reference group.

Random effects adjustments were made for age, daily income earnings, cigarette smoking, biomass burning, marital status, work duration and religion.

### Effects of metal exposures on hemoglobin levels

The exposure to metals such as Pb, Cd, Rb, Eu and Tb were associated with significant reductions in Hb levels in both the e-waste recyclers and the reference population (Table 5). When analysis was restricted to the recyclers only, significant reduction in Hb levels was still observed with increase in the blood level concentrations of Pb, Cd, Rb, Eu and Tb (see appendix F). Furthermore, the high concentrations of La, Y, Ce and Tl in blood was observed to be associated with significant increase in Hb level in both groups. Similarly, increases in levels of Y in blood was associated with significant increases in Hb levels of the recyclers.

Among comparator group, La levels in blood were found to be associated with significant increase in Hb levels (β = 0.063; 95% CI: 0.015, 0.111; *p* = *0.01*). However, higher levels of Tb in blood were associated with significant reductions in Hb (β= −0.095; 95% CI: −0.188, −0.002; *p* = *0.04*) levels among the comparison group.

Random effects adjustments were made for age, daily income earnings, cigarette smoking, biomass burning, marital status, work duration and religion.

### Nutrients and metal exposure interaction on hemoglobin levels

In the joint effect model, a unit increase in Fe level, leads to 0.20 g/dL increase in Hb levels (95% CI: 0.037, 0.368; *p* = *0.02*; Appendix G) after Pb exposure. Particularly among the recyclers, 1 mg of Fe consumed was associated with a 0.23 g/dL increase in Hb levels (95% CI: 0.013, 0.445; *p* = *0.04*; Appendix H). Furthermore, the consumption of Fe-rich diets significantly increased Hb levels in both groups (β = 0.164, 95% CI: 0.001, 0.327; *p* = *0.04*; Appendix H) exposed to Cd.

Although the recyclers were highly exposed to a wide diversity of metals; including, Pb, Cd, Tb, Rb and Eu at baseline, the consumption of Fe-rich diets was found to be associated with significant increase in Hb levels (Appendix H).

## Discussion

In our current study, we show that though long-term exposure to metals like Pb, Cd, Tb, Rb and Eu may reduce Hb levels, the consumption of Fe-and protein-rich diets may ameliorate the effect of metal-induced anemia among the e-waste recyclers.

### Chronic metal exposure

This study is arguably one of the first to measure the blood levels of metals in the lanthanide series (Eu, Tb, La, Tl, Sr, Ce, Rb, & Y) among informal e-waste recyclers overtime. While longitudinal studies on rare earth elements are scarce, there are few snapshot biomonitoring studies to make relevant comparisons with. Baseline mean concentrations of Ce and La detected in our reference population were higher than in the recyclers. In contrast, blood Ce and La levels in adult Romanians who smoke and/or use electronic cigarettes (0.31, 0.22 ng/mL, respectively) were higher than in the control group (Badea et al., 2018). In a pilot cross-sectional study, Badea, Luzardo (Badea et al., 2018) measured serum levels of Ce (0.39 ng/mL), Nd (0.26 ng/mL), and La (0.25 ng/mL), with the highest blood values in the 95th percentile. These levels were comparable to those seen in adult Romanians who smoke and/or use electronic cigarettes (Ce: 0.31, Nd: 0.30, and La: 0.22 ng/mL, respectively), but higher than those found in the control group (Badea et al., 2018). The baseline mean blood La (0.34 μg/L) levels among e-waste recyclers in the current study were substantially higher than those reported by Gaman, Delia (Gaman et al., 2020) and Badea, Luzardo (Badea et al., 2018), whereas Ce levels were much higher than the mean values we describe here. Again, blood levels of Sr documented among male automobile workers (1.5 ± 1.54 mg/L) and their controls (0.22 ± 0.26 mg/L) in Kolkata-India (Basu et al., 2014) were ten times higher than levels detected among e-waste recyclers in the current study. The exposure to higher levels of Ce and La has been found to play a crucial role in the induction and promotion of various types of cancer, including oral, lung, and colon cancer (Brouziotis et al., 2022; Rim et al., 2013). Additionally, exposure to La has been linked to the development of gastrointestinal issues, such as nausea, vomiting and diarrhea (Rothenberg et al., 2015). Further research is required to better understand human exposures to rare earth elements including Sr, Ce, Eu, La, Nd, Rb, Tb, and Y since we do not currently have blood reference ranges to compare results with. Beyond these, the decreased levels of Pb, Mn, and Rb among recyclers is possible. This is because, periodic shift from e-waste recycling to subsistence farming activities among recyclers might possibly have resulted in the observed reduction in levels of Pb, Mn, and Rb, given the inconsistency in exposure overtime.

Our discussion focus here will remain on metals that are of greatest health concern (Pb and Cd). In the current analyses, blood Pb measured among the recyclers, during the last two time points of the study, was relatively lower than the baseline (Takyi et al., 2021) levels as well as concentrations reported by Srigboh, Basu (Srigboh et al., 2016) (mean 79.3 mg/L) in e-waste recyclers at Agbogbloshie. Although the observed Pb exposure declined, levels reported overtime are still more than the recommended limits. Despite the fact that environmental exposure to Pb has significantly reduced in recent years, constant levels of circulating Pb in blood can be attributed primarily to a continued exchange of Pb between the bones, blood and soft tissue due to the substantial difference in half-lives of Pb in various compartments (i.e., blood, tissue, and bone) (Camaj et al., 2020; Rădulescu and Lundgren, 2019; Tiesjema and Mengelers, 2017). More crucially, under certain conditions, Pb in bone can be mobilized at a faster rate and released back into the circulatory system to restore homeostasis (Camaj et al., 2020; Camaj et al., 2018). Accordingly, the observed lower Hb levels among the recyclers (especially at baseline and time point two), may be associated with current and cumulative exposures.

Among the reference population, blood Pb and Cd concentrations significantly reduced overtime, suggesting seasonal variations as a key predictor of metal exposures. Among the reference group, the levels of metals such as Pb and Cd measured were within the ranges reported during the 2013–2014 cycle of the U.S. National Health and Nutrition Examination Survey (NHANES) and were around the 90th percentile value.

### Prevalence of anemia

“Nutritional anaemias” result when the intake of key nutrients is inadequate to meet the demands for the synthesis of Hb and RBCs. The prevalence of anemia, especially due to nutrient deficiency, is very high in developing countries. The current study’s findings of low Hb levels observed among the e-waste recyclers revealed the prevalence of Pb-induced anemia. Although no clear-cut reference limits exist for groups exposed to toxic metals, the WHO defines anemia in males as having an Hb level of less than 13 g/dL (32). Among Chinese adult cohort exposed to toxic metals, anemia was highly prevalent, with 28.1% meeting the WHO criterion (Elbarbary et al., 2020). We did not find significant differences in Hb levels measured in the recyclers and referent population, given the small sample size used in this study.

Several studies have documented contrary reasons for the effects of physical activity on Hb levels in humans. For instance, while some researchers relate physical activity to exercise-induced haemolysis (Hu and Lin, 2012; Çiçek, 2018), others have also reported the positive effects of exercise on Hb levels (Jannah, 2020). Here, we measured higher Hb levels among individuals of the reference population who were generally sedentary; hence, the effects of physical activity and Hb levels remain inconclusive.

Our findings have a number of policy ramifications. First, we have demonstrated that anemia among e-waste recyclers is a significant public health concern in Ghana, given that more than half of recyclers had some degree of anemia. Nonetheless, anemia among groups exposed to toxic metals like e-waste recyclers has received no attention from both a scientific and policy standpoint. Second, we found a significant difference in the incidence of anemia between e-waste recyclers and a reference group, which can help direct resources and programs for groups exposed to toxic chemicals in Ghana and elsewhere.

### Nutrients & anemia

The findings demonstrate that anemia associated nutrient in-adequacies (Ca, Cu, Se, Mg, Vitamins A, B_12_, C & D) (SA Takyi et al., 2020) are common among both e-waste recyclers and the reference population as per the set RDA guidelines. These findings further suggest that the deficiency of multiple nutrients, as opposed to a single nutrient inadequacy, accompanied with increased persistence of anemia. Our participants were nutritionally deficient, given their low socioeconomic status (SA Takyi et al., 2020).

Moreover, we also observed that dietary intake of Fe-rich diets was associated with significant increase in Hb levels in both groups as well as when analysis was restricted to only the e-waste recyclers. Probably, the increased intake of green leafy vegetables among both recyclers and reference group is likely to have contributed to the higher dietary Fe adequacy, considering the nature of their staple foods.

Several studies have documented the positive implications of dietary protein intake on anemia incidence (Kokubo et al., 2016; Aritonang and Siagian, 2017; Elba et al., 2021). Similarly, we found dietary intake of protein to be associated with increase in Hb levels among both groups as well as when analysis was restricted to the recyclers. These consistent results may be explained by the function that protein plays in hemopoiesis, or the process of making blood cells and platelets, as well as in the body’s transfer of iron (Muckenthaler et al., 2017; Shvartsman et al., 2019). Anemia may result from an interruption in Fe transport once the protein consumption pattern is inadequate.

### Metals and anemia

Toxic metals commonly affect certain organs and molecular targets, such as the bone marrow’s hemopoietic system, which can result in anemia. For instance, Pb can occupy Fe ion sites in the intestinal mucosa or hematological system, thus boosting absorption or reduction in Pb excretion (Wang et al., 2021); thus potentiating the likelihood of anemia occurrence.

The impact of metal exposures on Hb levels among e-waste recyclers and a reference population are consistent with the results of recent Chinese and some experimental studies (Peters et al., 2021; Horiguchi et al., 2011; Chen et al., 2015). For instance, Chen, Zhou (Chen et al., 2015) reported that Cd and Pb may have interactive effects on Hb. The high levels of Pb in the blood binds to RBCs, impairing the synthesis of “heme,” which is critical to life; as it transports oxygen to all body tissues. In addition, the critical effects of Pb in the body may be attributed to its interference with several enzymes involved in heme synthesis. The interference includes the inhibition of aminolevulinic acid dehydratase (ALAD) and changes in some metabolite concentrations (e.g., deltaaminolevulinic acid in the urine (ALA-U), coproporphyrin in urine (CP), and zinc protoporphyrin (ZPP) (Pachathundikandi and Varghese, 2006; Patrick, 2006; R Rahimpoor et al., 2020). For that reason, these enzymes may be considered as markers of Pb exposure. In addition, the exposure to Pb may shorten the longevity of red blood cells (RBCs), increasing the risks of anemia (Son and Yang, 2019; Ukaejiofo et al., 2009). These may imply that anemia induced by chronic Pb exposure may be triggered by both interference with heme biosynthesis and decreased RBC survival. In view of this, Pourabdian, EIZADI (Pourabdian et al., 2011) had postulated that biomarkers of Hb, mean corpuscular volume (MCV), and white blood cell (WBC) were not dose-dependent on blood Pb levels. The inconsistence in opinions may be due to differences in dietary intake patterns of the subject populations studied. In addition, the inconsistency of the findings may be a reflection of a number of other reasons. First, all of these studies had a small geographically unique and so non-representative sample size, thus limiting the generalizability of the findings. Next, short-term metal exposure may likely influence the hematologic and haematopoietic physiology differently compared to the long-term exposure (Elbarbary et al., 2020). Lastly, older persons may be impacted differently than younger people.

More importantly, the observation that exposure to emerging metals like Eu, Tb and Rb appears to reduce Hb levels in humans is noteworthy. Similarly, Henríquez-Hernández, Boada (Henríquez-Hernández et al., 2017) findings suggest that the exposure to Eu, which is commonly found in e-waste, may have subtle effects on the Hb levels of sub-Saharan immigrants. The scant details on how the exposure to these emerging metals influence Hb levels necessitates detailed future studies to identify and substantiate the biological plausibility of such findings.

### Metals, diet & anemia

There is a rich body of evidence that Fe-rich diet intake plays a key role in Pb poisoning (Engwa et al., 2019; Kwong et al., 2004; Mitra et al., 2017; Słota et al., 2022; Kim et al., 2003) pre-existing Fe-deficiency has been linked to an increased risk of exposure to Pb; including that its absorption and subsequent metal poisoning has been reported in several previous investigations (Słota et al., 2022; Wright et al., 2003). As mentioned earlier, Fe deficiency has been reported to lead to alterations in Divalent Metal (Ion) Transporter 1 (DMT1) expression, which appear to augment Pb absorption rates in the gastro-intestinal (GI) tract and thus, increasing the risk and development of environmental exposure-induced anemia. Here, while the recyclers were heavily exposed to a myriad of metals, including Pb, Cd, Tb, Rb, and Eu, our findings seem to suggest that their consumption of Fe-rich diets was associated with significant increases in Hb levels. Predictively, Fe-rich diet intake may mitigate the effects of a wide range of metals prevalent in the environment by lowering their absorption and perhaps increasing their removal from the body; thus may improve Hb levels overtime. Further substantiating our findings, while the exposure to Pb may shorten the longevity of RBCs, (hence reduce Hb levels) (Son and Yang, 2019; Ukaejiofo et al., 2009), several authors have cited Fe-rich dietary intake to boost the production of Hb and further dampen the effects of Pb in humans (Słota et al., 2022; Gundacker et al., 2021). Most studies generally report similar but less consistent findings among children, women or general population (Hsieh et al., 2017; Słota et al., 2022; Hegazy et al., 2010; Kim 2018; Kim and Park, 2014; Turgut et al., 2007). They either reported the relationship between metal exposures and anemia or dietary Fe intake and anemia. Our study is the first to indicate a joint relationship between dietary Fe-intake, metal exposure (Pb, Cd, Tb, Rb, and Eu) and Hb among e-waste recyclers and referent population. However, further mechanistic studies are required to better understand the joint effects and pathways through which dietary Fe may concurrently reduce the effects of metals and further improve Hb levels in humans.

### Limitations and strengths

Given that the study relied on participants’ self-reported 48-hour food recalls to determine nutrient intake from meals taken, the results gathered may be subject to errors due to recall bias. Dietary evaluations are largely pseudoscientific and therefore subject to recall bias, such as under- or mis-reporting of meal quantities taken. Although the prevalence of anemia is relatively high in developing countries like Ghana, we may not have explicitly considered all hematological parameters like haematocrit, RBCs, erythropoietin, total iron binding capacity, transferrin saturation and serum ferritin. For this reason, we adjusted for characteristics that were closely related or served as risk factors of anemia in the multivariable regression models. Given financial and logistical constraints, we could not obtain information on participant’s genetic susceptibility, despite the fact that genetic variants of Fe-related genes can play a role in the uptake of some of the metals. Further, physical activity levels of the e-waste recyclers and the referent population was not assessed. Notwithstanding these limitations, the study had some distinct and unique strengths and contributions. We therefore believe that the influence of the aforementioned limitations was largely compensated by the more reliable, stable and objective approach employed to quantify metals (Kutscher et al., 2018; Laur et al., 2020) and Hb levels (Bansal et al., 2016) in whole blood. We believe also that this work is relevant, since it is the first to investigate the joint effects of several metals, including emerging metals, and individual dietary nutrient intake on Hb levels of e-waste recyclers in a natural setting for nearly two years. In addition, daily dietary nutrient intake from whole foods rather than supplements was estimated.

## Conclusion

The low Hb levels among e-waste recyclers, was compensated for by the intake of Fe-rich diets, despite their higher exposure to several metals during informal e-waste recycling. Considering the fact that dietary Fe intake boosts blood Hb levels in e-waste recyclers, the Ministry of Health may need to establish nutrition programs targeted at highly chemical-exposed worker groups. Finally, future studies should focus on the integrative mechanisms of dietary Fe and composite metal absorption, as well as their impact on not only Hb levels but also on the levels of other hematological parameters like haematocrit levels, RBCs, erythropoietin, total iron binding capacity, transferrin saturation and serum ferritin among highly exposed workers.

## Figures and Tables

**Table 1:** Concentrations of metals in blood of e-waste recyclers and reference population overtime.

Metals (μg/L)	E-waste Recyclers		Reference Population		*p*-value
	
	Mean±SD	Median (IQR)	Mean±SD	Median (IQR)	
**Time Point 1**					
**Pb**	**92.35 ± 63.69**	76.82 (49.37)	40.67 ± 19.12	40.25 (17.45)	<0.01
**Cd**	0.73 ± 0.55	0.59 (0.43)	**0.93 ± 0.64**	0.81 (0.55)	<0.01
**Mn**	12.74 ± 5.09	12.56 (7.15)	**15.54 ± 5.16**	14.71 (7.69)	<0.01
**Rb**	2497.59 ± 573.80	2525.53 (691.85)	2561.96 ± 659.09	2543.11 (992.71)	0.60
**Ce**	0.16 ± 0.19	0.10 (0.08)	**0.47 ± 0.85**	0.06 (0.12)	<0.01
**Sr**	**50.25 ± 18.65**	48.84 (23.24)	41.56 ± 12.48	38.90 (19.12)	<0.01
**Eu**	0.018 ± 0.006	0.015 (0.005)	**0.023 ± 0.010**	0.020 (0.005)	<0.01
**Y**	0.05 ± 0.05	0.03 (0.03)	**0.13 ± 0.19**	0.05 (0.06)	<0.01
**La**	0.51 ± 0.59	0.34 (0.27)	0.61 ± 0.78	0.27 (0.21)	0.06
**Nd**	0.09 ± 0.06	0.07 (0.05)	0.20 ± 0.32	0.07 (0.04)	0.1
**Tl**	**1.81 ± 0.56**	1.73 (0.69)	1.14 ± 0.30	1.09 (0.24)	<0.01
**Tb**	0.01 ± 0.005	0.01 (0.005)	**0.02 ± 0.01**	0.02 (0.003)	<0.01
**Time Point 2**					
**Pb**	**76.80 ± 55.70**	67.18 (35.87)	31.23 ± 10.50	29.04 (19.36)	<0.01
**Cd**	0.73 ± 0.79	0.45 (0.51)	0.54 ± 0.24	0.49 (0.24)	0.60
**Mn**	6.70 ± 6.39	5.79 (3.69)	7.63 ± 7.05	6.45 (5.13)	0.45
**Rb**	1305.92 ± 246.23	1249 (386.31)	1264.63 ± 254.82	1208 (354.75)	0.24
**Ce**	0.27 ± 0.14	0.26 (0.15)	**1.22 ± 0.26**	1.16 (0.15)	<0.01
**Sr**	48.80 ± 28.93	42.70 (17.73)	42.53 ± 30.36	35.94 (14.83)	<0.01
**Eu**	0.009 ± 0.004	0.01 (0.005)	0.009 ± 0.006	0.008 (0.005)	0.28
**Y**	0.10 ± 0.03	0.11 (0.03)	**0.12 ± 0.07**	0.1 (0.025)	<0.01
**La**	**0.69 ± 0.17**	0.68 (0.05)	0.60 ± 0.24	0.54 (0.09)	<0.01
**Nd**	0.07 ± 0.05	0.05 (0.04)	0.10 ± 0.16	0.06 (0.06)	0.08
**Tl**	**2.39 ± 0.54**	2.32 (0.64)	1.98 ± 0.40	1.90 (0.43)	<0.01
**Tb**	0.006 ± 0.002	0.005 (0.001)	0.006 ± 0.004	0.005 (0.001)	0.54
**Time Point 3**					
**Pb**	**77.54 ± 47.59**	67.74 (33.26)	29.24 ± 9.52	27.92 (10.76)	<0.01
**Cd**	**0.73 ± 0.83**	0.50 (0.50)	0.41 ± 0.24	0.40 (0.20)	0.02
**Mn**	14.07 ± 15.90	8.58 (8.85)	17.75 ± 33.05	9.36 (9.58)	0.75
**Rb**	1174.76 ± 522.30	1276.52 (733.76)	1326.63 ± 374.40	1336.13 (322.11)	0.20
**Ce**	**2.02 ± 0.31**	1.95 (0.20)	0.85 ± 0.65	0.63 (0.16)	<0.01
**Sr**	**52.62 ± 18.54**	48.7 (18.1)	38.03 ± 8.88	38.25 (12.5)	<0.01
**Eu**	**0.022 ± 0.053**	0.015 (0.005)	0.015 ± 0.022	0.01 (0.003)	<0.01
**Y**	**0.28 ± 0.59**	0.14 (0.08)	0.19 ± 0.61	0.08 (0.03)	<0.01
**La**	1.25 ± 0.83	1.82 (1.60)	**5.39 ± 21.52**	1.86 (0.15)	0.02
**Nd**	**0.22 ± 0.20**	0.17 (0.08)	0.16 ± 0.37	0.08 (0.05)	<0.01
**Tl**	**2.16 ± 0.36**	2.20 (0.50)	1.77 ± 0.25	1.70 (0.30)	<0.01
**Tb**	**0.012 ± 0.007**	0.01 (0.01)	0.009 ± 0.013	0.005 (0.005)	<0.01

**Table 2:** Self-reported dietary nutrient intake of e-waste recyclers and the reference population.

Nutrients	E-waste Recyclers			Reference Population		
		
	RDA	Mean ± SD	Median (IQR)	Mean ± SD	Median (IQR)	*p*-value
			**Time Point 1**			
Folate (mcg)	400	42.21 ± 24.28	40 (31.01)	37.36 ± 26.62	27.75 (28.65)	0.26
Vitamin A (mcg)	900	527.68 ± 619.59	226.93 (816.75)	**942.32 ± 893.82**	706.8 (1258)	<0.01
Vitamin B_12_ (mcg)	2.4	0.54 ± 0.73	0.36 (0.40)	0.64 ± 0.66	0.48 (0.48)	0.45
Vitamin C (mg)	90	63.05 ± 49.02	45.58 (51.9)	63.22 ± 43.76	48.80 (43.15)	0.98
Vitamin D (mg)	15	0.49 ± 0.89	0.30 (0.40)	0.43 ± 0.55	0.28 (0.26)	0.64
Proteins (g)	56	72.15 ± 29.89	67.98 (33.83)	65.10 ± 26.96	59.55 (37.70)	0.16
Carbohydrates (g)		305.28 ± 105.93	295.00 (124.5)	287.53 ± 118.76	270.00 (179.00)	0.35
			**Time Point 2**			
Folate (mcg)	400	44.63 ± 30.37	40.03 (34.90)	40.65 ± 30.44	30.23 (42.66)	0.45
Vitamin A (mcg)	900	461.47 ± 585.12	168.50 (608.00)	**865.48 ± 884.25**	446.15 (1144.50)	<0.01
Vitamin B12 (mcg)	2.4	0.51 ± 0.61	0.37 (0.39)	0.70 ± 0.73	0.48 (0.57)	0.12
Vitamin C (mg)	90	49.73 ± 36.07	40.00 (40.1)	60.02 ± 44.02	48.35 (51.50)	0.12
Vitamin D (mg)	15	0.47 ± 0.97	0.28 (0.30)	0.72 ± 1.29	0.34 (0.52)	0.19
Proteins (g)	56	67.86 ± 24.75	64.60 (25.50)	65.72 ± 28.80	60.1 (27.15)	0.63
Carbohydrates (g)		**280.64 ± 82.88**	272.00 (117.50)	249.23 ± 67.79	242.00 (73.00)	0.02
			**Time Point 3**			
Folate (mcg)	400	44.12 ± 43.34	36.20 (37.05)	47.47 ± 27.39	46.03 (30.35)	0.64
Vitamin A (mcg)	900	535.15 ± 684.53	218.00 (612.10)	**857.27 ± 881.66**	485.00 (1152.50)	0.02
Vitamin B12 (mcg)	2.4	0.51 ± 0.78	0.24 (0.51)	0.52 ± 0.52	0.35 (0.36)	0.99
Vitamin C (mg)	90	71.38 ± 72.55	54.35 (55.24)	69.43 ± 50.09	54.15 (73.00)	0.87
Vitamin D (mg)	15	0.36 ± 0.39	0.27 (0.25)	0.39 ± 0.19	0.34 (0.19)	0.73
Proteins (g)	56	89.51 ± 230.31	64.60 (30.50)	66.71 ± 25.29)	59.43 (28.73)	0.51
Carbohydrates (g)		**301.72 ± 88.50**	299.00 (114.50)	265.56 ± 84.26	263.00 (113.75)	0.02

Footnote: Protein was based on average body weight of study participants (69.5 kg).

p-value notation: *p*<0.05 for which reason their values were boldened.

**Table 3:** Prevalence of Anemia among e-waste recyclers and reference population.

	E-waste Recycler n (%)	Reference Group n (%)	χ2	*p*-value
**Time Point 1**				
			0.48	0.49
Non-anemic	47 (47.00)	27 (52.94)		
Anemic	53 (53.00)	24 (47.06)		
**Time Point 2**				
			0.09	0.77
Non-anemic	111 (78.17)	52 (80.00)		
Anemic	31 (21.83)	13 (20.00)		
**Time Point 3**				
			1.36	0.24
Non-anemic	126 (88.73)	53 (82.81)		
Anemic	16 (11.27)	11 (17.19)		

Hemoglobin (g/dL) concentrations to diagnose anemia among e-waste recyclers and the reference population.

**Table 4:** Relationship between nutrient intake and Hemoglobin levels among e-waste recyclers and reference population.

Nutrients	Hb g/dL β [95% CI]	p-value
Fe	0.208* [0.051, 0.366]	0.01
Mg	− 0.011 [−0.085, 0.139]	0.59
Zn	0.027 [−0.093, 0.129]	0.64
Ca	0.062 [−0.035, 0.160]	0.21
Se	−0.025 [−0.059, 0.009]	0.14
Cu	0.026 [−0.036, 0.088]	0.41
Folate	0.013 [−0.039, 0.066]	0.63
Vit A	0.010 [−0.018, 0.038]	0.47
Vit B12	0.020 [−0.034, 0.075]	0.46
Vit C	0.010 [−0.032, 0.053]	0.64
Vit D	− 0.011 [−0.049, 0.026]	0.55
Proteins	0.155* [0.002, 0.309]	0.04
CHO	0.097 [−0.038, 0.232]	0.16

* *p*-value notation: *p*<0.05 for which reason their values were boldened.

**Table 5:** Effects of metal exposures on hemoglobin levels among e-waste recyclers and reference population.

Metals	Hb g/dL β [95% CI]	p-value
B-Pb	−0.1358* [−0.241, −0.029]	0.01
B-Cd	−0.088* [−0.152, −0.025]	<0.01
B-Mn	− 0.012 [−0.039, 0.016]	0.39
B-Rb	−0.064* [− 0.114, − 0.015]	<0.01
B-Sr	0.024 [−0.003, 0.051]	0.08
B-Y	0.032* [0,003, 0.062]	0.03
B-La	0.057* [0.025, 0.089]	<0.01
B-Ce	0.024* [0.007, 0.041]	<0.01
B-Nd	−0.002 [−0.036, 0.032]	0.91
B-Eu	−0.066* [−0.107, −0.025]	<0.01
B-Tb	−0.085* [−0.134, −0.037]	<0.01
B-Tl	0.039* [0.003, 0.075]	0.04

* *p*-value notation: *p*<0.05 for which reason their values were boldened. Random effects adjustments were made for age, daily income earnings, cigarette smoking, biomass burning, marital status, work duration and religion.

## APPENDIX

**Table A:** Changes in metal levels from March 2017 to April 2018 among e-waste recyclers and reference group.

Metal Levels	E-waste Recyclers β [95% CI]	Comparison Group β [95% CI]
**Pb**	**−0.194*** [−0.374, − 0.013]	**−0.676**** [−1.002, −0.349]
**Cd**	− 0.043 [−0.167, 0.081]	**−0.822**** [−1.013, −0.630]
**Mn**	**−0.128*** [−0.231, − 0.024]	**−0.230**** [−0.349, −0.111]
**Rb**	**−0.995**** [−1.123, −0.868]	**-1.204**** [−1.436, −0.973]
**Ce**	**0.554**** [0.524, 0.584]	**0.323**** [0.245, 0.401]
**Sr**	0.144 [−0.097, 0.386]	− 0.218 [−0.608, 0.172]
**Eu**	− 0.121 [−0.276, 0.033]	**−0.536**** [−0.709, −0.364]
**Y**	**0.693**** [0.624, 0.763]	0.139 [−0.025, 0.303]
**La**	**0.599**** [0.484, 0.713]	**0.601**** [0.502, 0.701]
**Nd**	**0.538**** [0.435, 0.640]	−0.080 [−0.231, 0.071]
**Tl**	**0.880**** [0.581, 1.178]	**1.152**** [1.194, 1.847]
**Tb**	− 0.049 [−0.231, 0.133]	**−0.725** **[−0.906, −0.545]

*p*-value notation:

* *p*<0.05

** *p*<0.01 for which reason their values were boldened.

**Table B:** Changes in nutrient intake March 2017 to April 2018 among e-waste recyclers and reference population.

Nutrient	E-waste Recyclers β [95% CI]	Reference Population β [95% CI]
Folate (mg)	−0.047 [−0.151, 0.058]	0.107 [−0.007, 0.221]
Vitamin A (mcg)	− 0.013 [−0.086, 0.060]	−0.050 [−0.160, 0.059]
Vitamin B_12_ (mcg)	−0.055 [−0.166, 0.056]	−0.039 [−0.191, 0.113]
Vitamin C (mg)	0.046 [−0.068, 0.159]	−0.0002 [0.174, 0.174]
Vitamin D (mg)	−0.065 [−0.174, 0.044]	0.109 [−0.055, 0.274]
Proteins (g)	−0.039 [−0.241, 0.164]	0.053 [−0.288, 0.394]
Carbohydrates (g)	0.015 [−0.250, 0.279]	−0.108 [−0.477, 0.261]

**Table C:** Mean Hb levels of e-waste recyclers and reference population overtime.

Time Point	Overall Hb level of participants (g/dL) Mean ± SD	E-waste recyclers Hb level (g/dL) Mean ± SD	Reference Population Hb level (g/dL) Mean ± SD	*p*-value
Time 1	13.00 ± 2.93	12.99 ± 3.17	13.02 ± 2.37	0.35
Time 2	13.99 ± 2.18	13.90 ± 2.15	14.16 ± 2.27	0.20
Time 3	14.56 ± 2.36	14.72 ± 2.48	14.08 ± 2.00	0.06

**Table D:** Comparison of measured Hb levels between e-waste recycler groups.

Hb levels (g/dL)	Burners	Dismantlers	Sorters / Collectors	p-value

	Mean ± SD	Mean ± SD	Mean ± SD	
**Time 1**	14.74 ± 3.30	12.31 ± 2.97	11.76 ± 2.19	<0.01
**Time 2**	13.03 ± 2.31	14.06 ± 2.19	14.46 ± 1.53	0.10
**Time 3**	14.71 ± 1.85	14.40 ± 2.25	15.65 ± 1.45	0.03

**Table E:** Relationship between nutrient intake and hemoglobin levels among e-waste recyclers.

Nutrients	Hb g/dL β [95% CI]	p-value
Fe	**0.266** [0.066, 0.466]	0.01*
Mg	− 0.029 [−0.075, 0.016]	0.20
Zn	0.085 [−0.053, 0.223]	0.23
Ca	0.048 [−0.070, 0.167]	0.42
Se	**−0.045** [−0.084, 0.007]	0.02*
Cu	0.040 [−0.028, 0.108]	0.24
Folate	0.003 [−0.058, 0.063]	0.93
Vit A	0.013 [−0.019, 0.046]	0.41
Vit B12	0.020 [−0.065, 0.070]	0.95
Vit C	− 0.010 [−0.052, 0.045]	0.88
Vit D	− 0.020 [−0.064, 0.024]	0.55
Proteins	**0.212** [0.032, 0.391]	0.02*
CHO	0.113 [−0.046, 0.272]	0.16

*p*-value notation:

* *p*<0.05

** *p*<0.01 for which reason their values were boldened.

**Appendix F:** Effects of metal exposures on hemoglobin levels among e-waste recyclers.

Metals	Hb g/dL β [95% CI]	p-value
B-Pb	**−0.126** [−0.250, −0.002]	0.04
B-Cd	**−0.114** [−0.190, −0.038]	<0.01*
B-Mn	**−0.038** [−0.072, −0.003]	0.03
B-Rb	**−0.063** [−0.118, −0.007]	0.03
B-Sr	0.027 [−0.004, 0.058]	0.09
B-Y	0.027 [−0.006, 0.060]	0.11
B-La	**0.057** [0.013, 0.100]	0.01
B-Ce	0.020 [−0.001, 0.041]	0.06
B-Nd	−0.005 [−0.043, 0.034]	0.81
B-Eu	**−0.075** [−0.124, −0.025]	<0.01*
B-Tb	**−0.081** [−0.138, −0.023]	<0.01*
B-Tl	0.029 [−0.018, 0.077]	0.23

*p*-value notation:

* *p*<0.05

** *p*<0.01 for which reason their values were boldened.

**Table G:** Joint effect of metals and nutrients on hemoglobin levels of e-waste recyclers and reference population.

Metals & Nutrients	Hb g/dL β [95% CI]	p-value
B-Pb	−0.152 [−0.268, −0.036]	**0.01** *
Fe	0.202 [0.037, 0.368]	**0.02** *
B-Pb	−0.126 [−0.243, −0.008]	0.04*
Ca	0.052 [−0.050, 0.155]	0.31
B-Pb	−0.133 [−0.250, −0.016]	0.03*
Mg	−0.009 [−0.049, 0.031]	0.65
B-Pb	−0.129 [−0.246, −0.012]	0.03*
Cu	0.026 [−0.040, 0.091]	0.44
B-Pb	−0.132 [−0.249, −0.015]	0.03*
Zn	−0.010 [−0.132, 0.112]	0.87
B-Pb	−0.139 [−0.258, −0.020]	0.02*
Se	−0.011 [−0.048, 0.025]	0.54
B-Pb	−0.135 [−0.251, −0.018]	0.02*
Proteins	0.072 [−0.075, 0.219]	0.34
B-Pb	−0.128 [−0.246, −0.010]	0.03*
Vitamin A	0.005 [−0.024, 0.033]	0.75
B-Pb	−0.212 [−0.368, −0.055]	<0.01*
Vitamin B_12_	0.015 [−0.040, 0.071]	0.59
B-Pb	−0.134 [−0.251, −0.017]	0.02*
Vitamin C	−0.002 [−0.051, 0.046]	0.93
B-Pb	−0.126 [−0.250, −0.002]	0.04*
Vitamin D	−0.011 [−0.051, 0.030]	0.60
B-Pb	−0.122 [−0.245, −0.0001]	0.05
Folate	−0.004 [−0.059, 0.052]	0.89
B-Cd	−0.095 [−0.164, −0.025]	**<0.01** *
Fe	0.164 [0.001, 0.327]	**0.04** *
B-Cd	−0.097 [−0.168, −0.027]	<0.01*
Ca	0.059 [−0.042, 0.159]	0.25
B-Cd	−0.098 [−0.169, −0.028]	<0.01*
Mg	−0.012 [−0.053, 0.028]	0.56
B-Cd	−0.101 [−0.172, −0.031]	<0.01*
Cu	0.030 [−0.034, 0.094]	0.35
B-Cd	−0.103 [−0.173, −0.032]	<0.01*
Zn	−0.033 [−0.153, 0.088]	0.59
B-Cd	−0.100 [−0.171, −0.030]	<0.01*
Se	−0.006 [−0.042, 0.029]	0.72
B-Cd	−0.101 [−0.170, −0.031]	<0.01*
Proteins	0.080 [−0.064, 0.224]	0.28
B-Cd	−0.098 [−0.169, −0.028]	<0.01*
Vitamin A	0.00003 [−0.029, 0.029]	1.00
B-Cd	−0.126 [−0.213, −0.038]	<0.01*
Vitamin B_12_	0.030 [−0.027, 0.087]	0.30
B-Cd	−0.105 [−0.177, −0.033]	<0.01*
Vitamin C	−0.019 [−0.068, 0.031]	0.46
B-Cd	−0.092 [−0.166, −0.018]	0.02*
Vitamin D	−0.002 [−0.042, 0.038]	0.92
B-Cd	−0.094 [−0.166, −0.024]	<0.01*
Folate	−0.013 [−0.071, 0.044]	0.65
B-Rb	−0.101 [−0.163, −0.039	**<0.01** *
Fe	0.170 [0.005, 0.334]	**0.04**
B-Rb	−0.098 [−0.161, −0.035]	<0.01*
Ca	0.047 [−0.055, 0.149]	0.36
B-Rb	—101 [−0.164, −0.039]	<0.01*
Mg	−0.011 [−0.051, 0.029]	0.60
B-Rb	−0.102 [−0.165, −0.039]	<0.01*
Cu	0.031 [−0.035, 0.097]	0.35
B-Rb	−0.101 [−0.164, −0.038]	<0.01*
Zn	−0.010 [−0.132, 0.112]	0.87
B-Rb	−0.106 [−0.170, −0.042]	<0.01*
Se	−0.016 [−0.052, 0.021]	0.40
B-Rb	−0.110 [−0.173, −0.047]	<0.01*
Proteins	0.118 [−0.03, 0.268]	0.12
B-Rb	−0.103 [−0.165, −0.040]	<0.01*
Vitamin A	0.007 [−0.022, 0.036]	0.63
B-Rb	−0.090 [−0.170, −0.009]	0.03*
Vitamin B_12_	0.013 [−0.046, 0.072]	0.66
B-Rb	−0.101 [−0.164, −0.039]	<0.01*
Vitamin C	0.006 [−0.043, 0.054]	0.82
B-Rb	−0.104 [−0.170, −0.039]	<0.01*
Vitamin D	−0.001 [−0.041, 0.038]	0.95
B-Rb	−0.095 [−0.159, −0.032]	<0.01*
Folate	0.008 [−0.048, 0.064]	0.78
B-Eu	−0.072 [−0.119, −0.026]	**<0.01** *
Fe	0.172 [0.007, 0.337]	**0.04** *
B-Eu	−0.076 [−0.123, −0.029]	<0.01*
Ca	0.082 [−0.020, 0.184]	0.11
B-Eu	−0.073 [−0.120, −0.026]	<0.01*
Mg	−0.014 [−0.054, 0.027]	0.51
B-Eu	−0.074 [−0.121, −0.026]	<0.01*
Cu	0.035 [−0.031, 0.100]	0.30
B-Eu	−0.073 [−0.120, −0.026]	<0.01*
Zn	−0.031 [−0.153, 0.091]	0.62
B-Eu	−0.074 [−0.121, −0.026]	<0.01*
Se	−0.010 [−0.046, 0.026]	0.58
B-Eu	−0.072 [0.120, −0.025]	<0.01*
Proteins	0.052 [−0.097, 0.200]	0.49
B-Eu	−0.078 [−0.126, −0.031]	<0.01*
Vitamin A	0.010 [−0.019, 0.039]	0.50
B-Eu	−0.101 [−0.161, −0.041]	<0.01*
Vitamin B_12_	0.005 [−0.052, 0.062]	0.85
B-Eu	−0.075 [−0.123, −0.027]	<0.01*
Vitamin C	0.011 [−0.038, 0.060]	0.67
B-Eu	−0.069 [−0.118, −0.019]	<0.01*
Vitamin D	−0.008 [−0.048, 0.031]	0.68
B-Eu	−0.070 [−0.119, −0.023]	<0.01*
Folate	−0.004 [−0.060, 0.052]	0.89
B-Eu	−0.101 [−0.156, −0.046]	**<0.01** *
Fe	0.186 [0.022, 0.349]	**0.03** *
B-Tb	−0.102 [−0.157, −0.046]	<0.01*
Ca	0.082 [−0.019, 0.183]	0.11
B-Tb	−0.099 [−0.155, −0.043]	<0.01*
Mg	−0.014 [−0.054, 0.026]	0.48
B-Tb	−0.100 [−0.155, −0.044]	<0.01*
Cu	0.036 [−0.030, 0.101]	0.28
B-Tb	−0.098 [−0.154, −0.043]	<0.01*
Zn	−0.024 [−0.145, 0.097]	0.70
B-Tb	−0.102 [−0.158, −0.046]	<0.01*
Se	−0.016 [−0.052, 0.020]	0.39
B-Tb	−0.100 [−0.156, −0.044]	<0.01*
Proteins	0.042 [−0.106, 0.190]	0.58
B-Tb	−0.108 [−0.164, −0.052]	<0.01*
Vitamin A	0.011 [−0.018, 0.039]	0.47
B-Tb	−0.111 [−0.179, −0.043]	<0.01*
Vitamin B_12_	0.001 [−0.056, 0.058]	0.97
B-Tb	−0.104 [−0.160, −0.047]	<0.01*
Vitamin C	0.012 [−0.036, 0.061]	0.61
B-Tb	−0.093 [−0.152, −0.035]	<0.01*
Vitamin D	−0.011 [−0.051,0.028]	0.57
B-Tb	−0.098 [−0.155, −0.041]	<0.01*
Folate	−0.008 [−0.063, 0.048]	0.79

*p*-value notation:

* *p*<0.05

** *p*<0.01 for which reason their values were boldened.

**Table H:** Joint effect of metals and nutrients on hemoglobin levels of e-waste recyclers.

Metals & Nutrients	Hb g/dL β [95% CI]	p-value
B-Pb	−0.140 [−0.280, −0.0005]	**0.04** *
Fe	0.229 [0.013, 0.445]	**0.04** *
B-Pb	−0.118 [−0.260, 0.024]	0.10
Ca	0.043 [−0.078, 0.165]	0.48
B-Pb	−0.129 [−0.270, 0.013]	0.07
Mg	−0.019 [−0.068, 0.030]	0.44
B-Pb	−0.120 [−0.261, 0021]	0.10
Cu	0.034 [−0.039, 0.106]	0.36
B-Pb	−0.125 [−0.267, 0.016]	0.08
Zn	0.011 [−0.134, 0.157]	0.88
B-Pb	−0.139 [−0.282, 0.003]	0.05
Se	−0.026 [−0.067, 0.016]	0.22
B-Pb	−0.127 [−0.268, 0.013]	0.07
Proteins	0.054 [−0.111, 0.220]	0.52
B-Pb	−0.115 [−0.258, 0.027]	0.11
Vitamin A	0.003 [−0.031, 0.037]	0.84
B-Pb	−0.210 [−0.406, −0.014]	**0.04** *
Vitamin B_12_	0.0002 [−0.064, 0.065]	0.99
B-Pb	−0.125 [−0.265, 0.016]	0.08
Vitamin C	−0.013 [−0.069, 0.042]	0.63
B-Pb	−0.119 [−0.264, 0.025]	0.10
Vitamin D	−0.023 [−0.070, 0.024]	0.34
B-Pb	−0.106 [−0.250, 0.038]	0.15
Folate	−0.028 [−0.091, 0.035]	0.39
B-Cd	−0.098 [−0.182, −0.013]	**0.02** *
Fe	0.201 [−0.014, 0.417]	0.07
B-Cd	−0.106 [−0.191, −0.021]	**0.01** *
Ca	0.048 [−0.071, 0.166]	0.43
B-Cd	−0.106 [−0.191, −0.021]	**0.01** *
Mg	−0.026 [−0.075, 0.023]	0.30
B-Cd	−0.113 [−0.196, −0.028]	**<0.01** *
Cu	0.042[−0.029, 0.112]	0.24
B-Cd	−0.111 [−0.197, −0.025]	**0.01** *
Zn	−0.010 [−0.153, 0.134]	0.89
B-Cd	−0.106 [−0.191, −0.021]	**0.01** *
Se	−0.022 [−0.062, 0.019]	0.29
B-Cd	−0.110 [−0.194, −0.026]	**0.01** *
Proteins	0.064 [−0.098, 0.225]	0.44
B-Cd	−0.107 [−0.191, −0.023]	**0.01** *
Vitamin A	−0.003 [−0.037, 0.030]	0.84
B-Cd	−0.147 [−0.252, −0.042]	**<0.01** *
Vitamin B_12_	0.022 [−0.043, 0.088]	0.50
B-Cd	−0.121 [−0.207, −0.035]	**<0.01** *
Vitamin C	−0.037 [−0.093, 0.019]	0.20
B-Cd	−0.102 [−0.188, −0.015]	**0.02** *
Vitamin D	−0.012 [−0.058, 0.035]	0.61
B-Cd	−0.096 [−0.180, −0.011]	**0.03** *
Folate	−0.049 [−0.115, 0.018]	0.15
B-Rb	−0.106 [−0.179, −0.032]	**<0.01** **
Fe	0.224 [0.011, 0.438]	**0.04** *
B-Rb	−0.098 [−0.173, −0.023]	**0.01** *
Ca	0.039 [−0.081, 0.159]	0.52
B-Rb	−0.105 [−0.180, −0.031]	**<0.01** *
Mg	−0.026 [−0.075, 0.023]	0.30
B-Rb	−0.101 [−0.175, −0.027]	**<0.01** *
Cu	0.037 [−0.035, 0.110]	0.31
B-Rb	−0.102 [−0.177, −0.027]	**<0.01** *
Zn	0.026 [−0.118, 0.171]	0.72
B-Rb	−0.116 [−0.192, −0.041]	**<0.01** *
Se	−0.036 [−0.078, 0.006]	0.09
B-Rb	−0.113 [−0.187, −0.039]	**<0.01** *
Proteins	0.110 [−0.058, 0.278]	0.20
B-Rb	−0.104 [−0.176, −0.032]	**<0.01** *
Vitamin A	0.005 [−0.029, 0.039]	0.76
B-Rb	−0.074 [−0.173, 0.025]	0.14
Vitamin B_12_	−0.005 [−0.074, 0.065]	0.90
B-Rb	−0.101 [−0.174, −0.028]	**<0.01** *
Vitamin C	−0.005 [−0.060, 0.050]	0.87
B-Rb	−0.105 [−0.181, −0.029]	**<0.01** *
Vitamin D	−0.014 [−0.060, 0.033]	0.56
B-Rb	−0.092 [−0.166, −0.017]	**0.02** *
Folate	−0.019 [−0.082, 0.043]	0.54
B-Eu	−0.079 [−0.134, −0.024]	**<0.01** **
Fe	0.214 [0.001, 0.428]	**0.04** **
B-Eu	−0.081 [−0.137, −0.025]	**<0.01** *
Ca	0.074 [−0.046, 0.193]	0.22
B-Eu	−0.081 [−0.137, −0.025]	**<0.01** *
Mg	−0.026 [−0.075, 0.023]	0.30
B-Eu	−0.081 [−0.137, −0.025]	**<0.01** *
Cu	0.047 [−0.025, 0.120]	0.20
B-Eu	−0.078 [−0.134, −0.022]	**<0.01** *
Zn	−0.012 [−0.156, 0.132]	0.87
B-Eu	−0.081 [−0.137, −0.025]	**<0.01** *
Se	−0.027 [−0.068, 0.014]	0.20
B-Eu	−0.078 [−0.134, −0.022]	**<0.01** *
Proteins	0.029 [−0.136, 0.195]	0.73
B-Eu	−0.085 [−0.141, −0.029]	**<0.01** *
Vitamin A	0.010 [−0.024, 0.043]	0.57
B-Eu	−0.117 [−0.189, −0.044]	**<0.01** *
Vitamin B_12_	−0.011 [−0.077, 0.055]	0.73
B-Eu	−0.078 [−0.135, −0.022]	**<0.01** *
Vitamin C	−0.002 [−0.058, 0.054]	0.95
B-Eu	−0.075 [−0.133, −0.017]	**0.01** *
Vitamin D	−0.024 [−0.071, 0.022]	0.30
B-Eu	−0.076 [−0.133, −0.019]	**<0.01** *
Folate	−0.028 [−0.091, 0.034]	0.37
B-Tb	−0.093 [−0.158, −0.028]	**<0.01** **
Fe	0.234 [0.020, 0.449]	**0.03** **
B-Tb	−0.090 [−0.156, −0.024]	**<0.01** *
Ca	0.073 [−0.047, 0.193]	0.23
B-Tb	−0.091 [−0.157, −0.025]	**<0.01** *
Mg	−0.027 [−0.076, 0.022]	0.28
B-Tb	−0.088 [−0.154, −0.023]	**<0.01** *
Cu	0.043 [−0.029, 0.116]	0.24
B-Tb	−0.086 [−0.151, −0.020]	**0.01** *
Zn	0.001 [−0.143, 0.145]	0.99
B-Tb	−0.094 [−0.161, −0.028]	**<0.01** *
Se	−0.031 [−0.072, 0.010]	0.14
B-Tb	−0.088 [−0.154, −0.022]	**<0.01** *
Proteins	0.030 [−0.136, 0.196]	0.72
B-Tb	−0.095 [−0.161, −0.029]	**<0.01** *
Vitamin A	0.008 [−0.026, 0.042]	0.65
B-Tb	−0.091 [−0.175, −0.008]	**0.03** *
Vitamin B_12_	−0.012 [−0.081, 0.057]	0.72
B-Tb	−0.089 [−0.155, −0.022]	**<0.01** *
Vitamin C	−0.003 [−0.059, 0.053]	0.91
B-Tb	−0.080 [−0.149, −0.012]	**0.02** *
Vitamin D	−0.025 [−0.071, 0.022]	0.30
B-Tb	−0.085 [−0.152, −0.017]	**0.01** *
Folate	−0.031 [−0.094, 0.032]	0.33

*p*-value notation:

* *p*<0.05

** *p*<0.01 for which reason their values were boldened.
